# Prehospital treatment with continuous positive airway pressure in patients with acute respiratory failure: a regional observational study

**DOI:** 10.1186/s13049-016-0315-3

**Published:** 2016-10-10

**Authors:** Vibe Maria Laden Nielsen, Jacob Madsen, Anette Aasen, Anne Pernille Toft-Petersen, Kenneth Lübcke, Bodil Steen Rasmussen, Erika Frischknecht Christensen

**Affiliations:** 1 Department of Anaesthesiology and Intensive Care Medicine, Aalborg University Hospital, Aalborg, Denmark; 2 Department of Clinical Medicine, Aalborg University, Aalborg, Denmark; 3 Prehospital Emergency Medical Services, North Denmark Region, Aalborg, Denmark

**Keywords:** Noninvasive ventilation, Continuous positive airway pressure, CPAP, Dyspnea, Respiratory insufficiency, Prehospital, Emergency medical services, Paramedic

## Abstract

**Background:**

Patients with acute respiratory failure are at risk of deterioration during prehospital transport. Ventilatory support with continuous positive airway pressure (CPAP) can be initiated in the prehospital setting. The objective of the study is to evaluate adherence to treatment and effectiveness of CPAP as an addition to standard care.

**Methods:**

In North Denmark Region, patients with acute respiratory failure, whom paramedics assessed as suffering from acute cardiopulmonary oedema, acute exacerbation of chronic obstructive pulmonary disease or asthma were treated with CPAP using 100 % O_2_ from 1 March 2014 to 3 May 2015. Adherence to treatment was evaluated by number of adverse events and discontinuation of treatment. Intensive care admissions and mortality were reported in this cohort. Effectiveness was evaluated by changes in peripheral oxygen saturation (SpO_2_) and respiratory rate during transport and compared to a historical control (non-CPAP) group treated with standard care only. Values were compared by hypothesis testing and linear modelling of SpO_2_ on arrival at scene and ΔSpO_2_ stratified according to treatment group.

**Results:**

In fourteen months, 171 patients were treated with CPAP (mean treatment time 35 ± 18 min). Adverse events were reported in 15 patients (9 %), hereof six discontinued CPAP due to hypotension, nausea or worsening dyspnoea. One serious adverse event was reported, a suspected pneumothorax treated adequately by an anaesthesiologist called from a mobile emergency care unit. Among CPAP patients, 45 (27 %) were admitted to an intensive care unit and 24 (14 %) died before hospital discharge. The non-CPAP group consisted of 739 patients. From arrival at scene to arrival at hospital, CPAP patients had a larger increase in SpO_2_ than non-CPAP patients (87 to 96 % versus 92 to 96 %, *p* < 0.01) and a larger decrease in respiratory rate (32 to 25 versus 28 to 24 breaths/min, *p* < 0.01). In a linear model, CPAP was superior to non-CPAP in patients with initial SpO_2_ ≤90 % (*p* < 0.05). One CPAP patient (0.6 %) and eight non-CPAP patients (1.1 %) were intubated in the prehospital setting.

**Discussion:**

The study design reflects the daily prehospital working environment including long transport timesand paramedics educated in treating symptoms of acute respiratory failure, rather than treating one specific diagnosis. The study population was included consecutively and few patients were lost to follow-up. However, the study was too small to allow assessment of any effect of prehospital CPAP on mortality, nor could the effectiveness in specific disease conditions be examined.

**Conclusions:**

In an emergency medical service including physician backup, adherence to CPAP treatment administered by paramedics was high and treatment was effective in patients with acute respiratory failure.

## Background

Acute respiratory failure is one of the major causes of emergency department admission as reflected by the fact that 7.3 % of calls to the Danish emergency medical communication centres (EMCCs) have “difficulty in breathing” as the main symptom [1, 2]. Common causes of non-traumatic breathing difficulties include acute cardiopulmonary oedema, lower respiratory tract infections and acute exacerbation of chronic obstructive pulmonary disease (COPD) or asthma, which in severe cases may require non-invasive positive pressure ventilation or endotracheal intubation and mechanical ventilation. Non-invasive positive pressure ventilation administered in hospital reduces mortality in patients suffering from acute cardiopulmonary oedema or acute exacerbations of chronic obstructive pulmonary disease, while its effect in patients with acute exacerbations of asthma is uncertain [3–7]. Patients with acute respiratory failure have a risk of further deterioration during transport to hospital by emergency medical services (EMS). To prevent situations of such deterioration, prehospital initiation of continuous positive airway pressure (CPAP) has become available. CPAP is administered to patients with spontaneous breathing through a non-invasive facemask with a positive airway pressure applied during the entire respiratory cycle and it has been shown that prehospital CPAP reduces dyspnoea and respiratory rate when compared to standard medical therapy alone [8, 9]. In previous studies of patients treated with CPAP in the prehospital setting, reporting of adverse events have included either none at all, mask intolerance or minor complications such as hypotension, nausea or vomiting [9–17]. In three of four randomised controlled trials in prehospital settings, standard care plus CPAP reduced intubation rate compared to standard care alone [9, 13, 14, 18]. There have been conflicting results in recent systematic reviews including both randomised and observational studies regarding the effectiveness of prehospital CPAP on mortality in patients with acute respiratory failure [19–21]. Original research studies of CPAP in Nordic EMS systems are sparse; in a Finnish observational study from 2003, use of prehospital CPAP administered by physicians to patients with presumed acute severe pulmonary oedema improved oxygen saturation, respiratory rate, heart rate and systolic blood pressure [15]. In 2014, the EMS organisation in North Denmark Region decided to let paramedics administer CPAP to patients with non-traumatic acute respiratory failure. Introduction of prehospital CPAP was preceded by educational training including preparation of a clinical guideline. The aim of this study is to evaluate 1) adherence to prehospital CPAP treatment in patients with acute respiratory failure and 2) effectiveness of additional CPAP in comparison with standard care only.

## Methods

### Study design

The present study was performed in two steps; firstly, a prospective study of adherence to prehospital CPAP treatment in patients with acute respiratory failure, administered in addition to standard care (subsequently denoted “CPAP group”). Secondly, in an uncontrolled before and after study, measures of effectiveness were compared to a historical “non-CPAP” group of patients with symptoms of acute respiratory failure in the same geographical area, identified from a database of prehospital medical records. The primary objective was to evaluate adherence to prehospital CPAP treatment assessed by incidence of adverse events and discontinuation of CPAP treatment. The secondary objective was to evaluate effectiveness of prehospital CPAP in a post-hoc analysis, assessed by changes in peripheral capillary oxygen saturation (SpO_2_) and in respiratory rate upon treatment.

### Setting

The regional EMS served 583,471 inhabitants in mixed rural and urban areas (7,933 km^2^) [22]. The EMS was structured as a three-tier system with 36 primary ambulances, five paramedic vehicles stationed in the rural areas and 24-h operative mobile emergency care units (MECUs) staffed by anaesthesiologists in two cities 54 km apart. On a daily basis, 130–150 patients were transported by the EMS to one of the three emergency departments in the region. Nurses operated the regional emergency medical communication centre by use of a Criteria Based Dispatch system, The Danish Index of Emergency Care [23]. Either the EMCC nurse or the EMS personnel on scene could request assistance from a MECU. Prior to the introduction of CPAP, paramedic supervisors completed a four-hour educational course on CPAP treatment conducted by an anaesthesiologist and a fellow doctor. The supervisors subsequently trained all paramedic squads for four hours. Additionally, comprehensive educational booklets on respiratory pathophysiology, indications, contraindications and practical handling were distributed to all paramedics including an overview action card. Whenever an EMCC nurse categorized a patient’s complaint as “difficulty in breathing”, one of the five paramedic vehicles equipped with disposable CPAP equipment was dispatched along with a primary ambulance. All EMS transport events were entered into a regional database, amPHI^TM^, which included registrations of the patient’s respiratory status observed upon EMS arrival, of treatments administered, and of clinical observations and vital signs obtained during transport.

### Participants

CPAP patients were included during the first 14 months after introduction of prehospital CPAP: 1 March 2014 to 3 May 2015. Selection criteria were all of the following three: 1) Patient age ≥18 years, 2) categorized by the EMCC nurse as having “difficulty in breathing” by use of The Danish Index of Emergency Care and 3) patient assessed by paramedics as suffering from acute exacerbation of COPD, asthma or acute cardiopulmonary oedema and treated with prehospital CPAP. Contraindications for initiation of CPAP treatment and reasons for discontinuing CPAP were as follows: Nausea or vomiting, reduced level of consciousness, respiratory fatigue, suspected pneumothorax on auscultation, hypotension (systolic blood pressure <90 mmHg), cranial or facial trauma, suspected stroke, foreign object in the airways, penetrating chest trauma, massive bleeding and epiglottitis.

In order to have a similar comparison group of adult patients with acute respiratory failure in the uncontrolled before and after study of effectiveness, we included non-CPAP patients from the regional EMS database, amPHI^TM^ from the previous 14 months prior to introduction of CPAP: 1 January 2013 to 28 February 2014. Non-CPAP selection criteria were all of the following three: 1) Patient age ≥18 years, 2) patient assessed by the arriving EMS personnel as having severe difficulty in breathing and 3) treated by the EMS with standard prehospital care which included high flow oxygen through a non-rebreathing facemask plus beta agonist and/or diuretics. In both study groups, pharmacological treatment was in some cases administered before arrival of the EMS, e.g. by the general practitioner who had called the EMCC. In both the CPAP and non-CPAP study groups, patients were included consecutively, including patients with repeated EMS transports during the intervention and control study periods.

### Standard prehospital care and intervention

Standard prehospital care for acute respiratory failure consisted of proper positioning of the patient, high flow oxygen administered by Bag-Valve-Mask ventilation (with 100 % oxygen at a flow of 6–12 L/min) and, depending on the presumed aetiology, salbutamol inhalation, intravenous furosemide, sublingual nitroglycerine spray and/or intravenous fentanyl. The MECU physician could be consulted by telephone and be called to the scene in case non-invasive ventilation was insufficient or in case of acute deterioration to secure optimal treatment including endotracheal intubation. In the CPAP study period, paramedics were instructed to administer CPAP immediately as an additional treatment to the standard care. The Flow-Safe II EZ® CPAP System (©Mercury Medical®, Clearwater, Florida, USA (CE 0086)) provided high flow oxygen on a fixed level of 100 % and adjustable CPAP/PEEP in the range 0–13 cm H_2_O [24]. An integral nebulizer allowed administration of medications without disrupting the positive pressure applied. CPAP treatment was continued until arrival at hospital if no adverse events occurred or contraindications arose.

### Variables and data collection

Prospective data collection included systematic recording of: Indication of CPAP treatment, the level of pressure applied, duration of CPAP treatment and any preterm discontinuation of CPAP, any adverse events, and any technical issues on a dedicated registration form. SpO_2_, systolic and diastolic blood pressures were monitored continuously by a LIFEPAK® 12-defibrillator/monitor (©Physio-Control, Inc., Redmond, Washington, USA). The prehospital database amPHI^TM^ provided information on demographics, paramedic assessment of the patient’s presumed cause of acute respiratory failure, pharmacological treatment, respiratory rate, endotracheal intubation, and transport times. For CPAP patients, number of days in hospital, intensive care unit (ICU) admission, in-hospital treatment with CPAP, non-invasive ventilation (NIV) or mechanical ventilation and mortality were collected from medical records via the regional Patient Administrative System. In the prospective study, the primary outcome measures were incidence of adverse events and discontinuation of CPAP treatment and secondary outcome measures included number of days in hospital, admissions to an ICU and in-hospital and 30 day mortality in CPAP patients. In the uncontrolled before and after study, changes in SpO_2_ and in respiratory rate measured upon arrival at scene and upon hospital arrival were primary outcome measures of effectiveness.

### Statistical methods

As the primary objective was adherence to an additional treatment, no power calculations were done prior to the study. Data was entered in EpiData Version 3.1. Statistics were calculated using “R” version 3.2.0. Descriptive data are presented as mean (± standard deviation (SD)) or median (interquartile range (IQR)). Vital signs were compared by either two-sample *t* test or Mann-Whitney test according to the distribution of data and results presented with 95 % confidence intervals [95 % CI]. Proportions were compared by chi-square test or Fisher’s exact test as appropriate. The association between SpO_2_ on arrival at scene and ΔSpO_2_ was modelled by linear regression (Generalized Linear Models (package glm)) with adjustment for sex and age and stratified according to CPAP or non-CPAP group. Patients were categorized according to initial SpO_2_ and recorded values ≤70 % were excluded from the linear model, as LIFEPAK12 did not provide precise measures of SpO_2_ below this limit, according to the manufacturer [25]. Linearity of the association between initial SpO_2_ value and ΔSpO_2_ were visually assessed and accepted, as was the normal distribution of residuals. The ggplot2 package (smoothing method LOESS) was used for graphical presentation. Two-tailed *p*-values below 0.05 were considered significant.

### Ethical approval

The Danish Data Protection Agency approved the study (record no. 2008-58-0028). According to The North Denmark Region Committee on Health Research Ethics, the study did not require ethical approval or collection of informed consent from the patients. The Danish Health Authority waived the requirement for patients to consent to have their medical records accessed by the researchers (3-3013-999/1/SABN).

## Results

### Adherence and adverse events related to prehospital CPAP treatment

Prehospital CPAP administration was adequately registered in 171 patients in the CPAP study period. Mean transport time from departure from scene to arrival at hospital was 27 (±12) min (*N* = 149, missing data in 22 patients). Paramedics administered CPAP treatment for ≤20 min in 42 cases, for 21–40 min in 78 cases and for more than 40 min in 48 cases and mean treatment time was 35 ± 18 min (*N* = 168, missing data in three patients). Adherence to treatment was 88 % (151/171) measured by the proportion of patients who continued CPAP treatment all the way to arrival at hospital. Reasons for discontinuation of CPAP treatment included intolerance to the facemask in eight patients, occurrence of an adverse event in six patients, prehospital intubation in one patient and unknown reasons in five patients. Overall, paramedics reported adverse events in 9 % (15/171) of the CPAP patients (Table 1).

**Table 1:** Adverse events of prehospital CPAP treatment

	CPAP (*N* = 171)
None	156
Hypotension^ab^	4
Hereof discontinued CPAP: 1	
Nausea	3
Hereof discontinued CPAP: 3	
Decrease in level of consciousness^c^	2
Hereof discontinued CPAP: 0	
Worsening dyspnoea	2
Hereof discontinued CPAP: 1	
Suspected pneumothorax	1
Hereof discontinued CPAP: 1	
Tachycardia	1
Coughing mucus	1
Dry nose and/or mouth	1

No missing data

*CPAP* continuous positive airway pressure

^a^Systolic blood pressure ≤90 mmHg

^b^None of the 4 cases were below 80 mmHg

^c^Glasgow Coma Scale from 15 to 14

In addition to the fifteen listed adverse events, five patients experienced a critical decrease in SpO_2_ during prehospital transport: from 92 to 86 %, from 97 to 91 %, from 87 to 80 %, from 93 to 85 % and from 94 to 71 %, respectively (*N* = 158, missing data in 13 patients). The latter of the cases was a patient with pulmonary sarcoidosis, in which SpO_2_ decreased markedly even before initiation of CPAP and a MECU was called to the scene. The attending anaesthesiologist suspected a pneumothorax and performed an ultrasound examination of the lungs. CPAP was discontinued and the anaesthesiologist performed a needle decompression, which resulted in audible escape of air from pleura, ease of breathing and immediate improvement in SpO_2_ from 71 % to 95 %. As previously described, discontinuation of CPAP before arrival at hospital due to an adverse event occurred in this and five more patients; three due to nausea, one due to hypotension and one due to worsening dyspnoea. The latter patient developed shallow breathing during transport, CPAP treatment was discontinued and replaced by Bag-Valve-Mask ventilation and the breathing pattern was normalised.

### In-hospital outcomes in CPAP treated patients

During hospital stay, 64 % (107/168) of patients in the CPAP group received no in-hospital ventilation support in the form of CPAP, NIV and/or mechanical ventilation. Twenty-seven percent of the patients (45/168) in the CPAP group were transferred to an ICU. Overall, in-hospital mortality was 14 % (24/168). Among ICU admitted patients, in-hospital mortality was 24 % (11/45) (Table 2).

**Table 2:** In-hospital outcomes in CPAP treated patients

	CPAP (*N* = 168)
Days in hospital, median (IQR)	5.5 (3.0–9.0)
Admitted to ICU, N (%)	45 (27 %)
Mortality	
In-hospital, all patients, N (%)	24 (14 %)
In-hospital, ICU patients, N (%)	11 (24 %)
30 days^a^, N (%)	36 (24 %)

No missing data

*CPAP* continuous positive airway pressure, *ICU* intensive care unit, *IQR* interquartile range

^a^Based on first time events only (*N* = 153)

The most frequent diagnoses among patients who died in hospital were acute exacerbation of COPD, lower airway tract infections and sepsis.

### Patient characteristics in CPAP versus non-CPAP groups in the prehospital setting

**Fig. 1:**
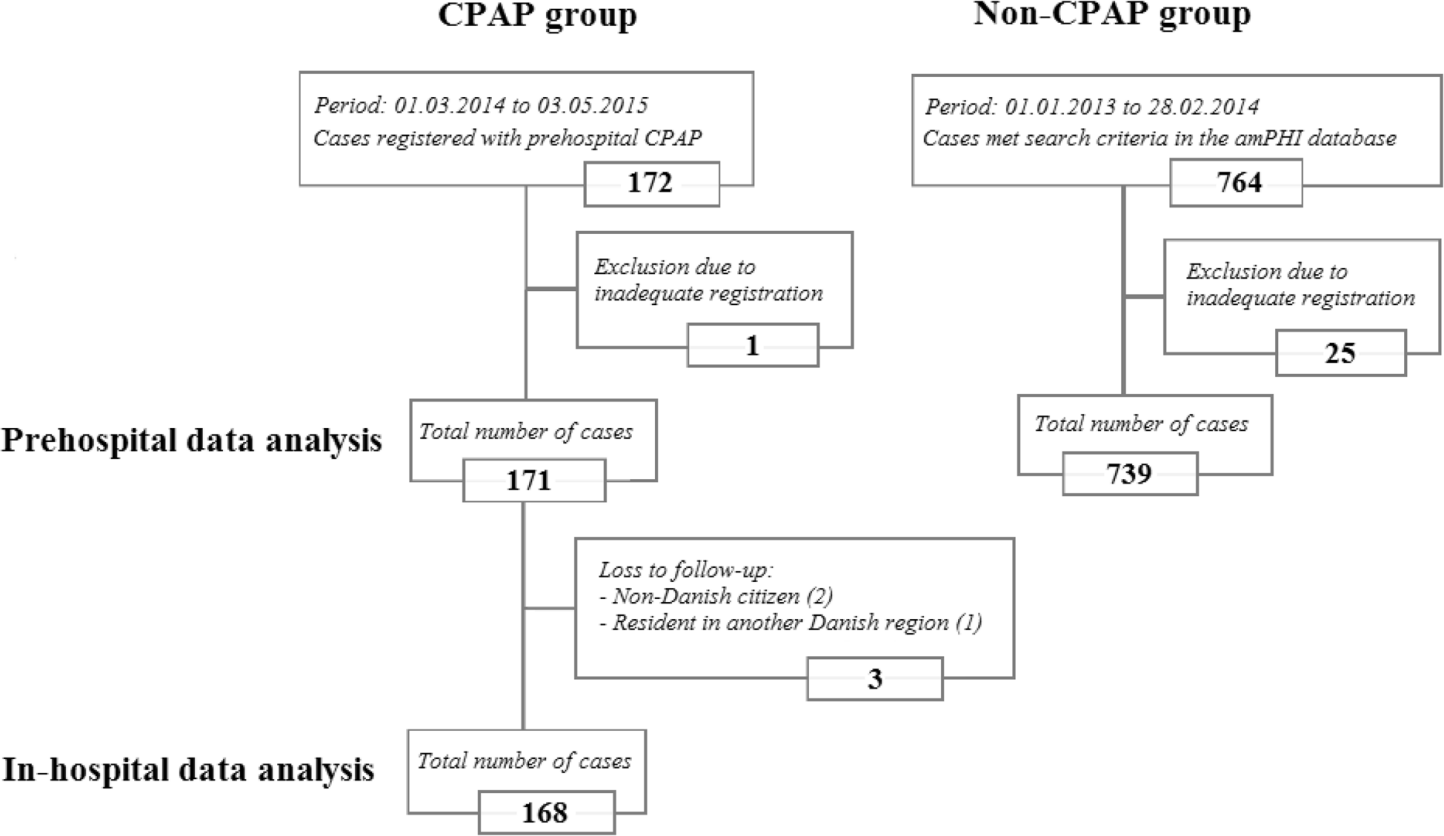
Study population flowchart

The CPAP group consisted of 171 patients and 739 patients met the criteria for inclusion in the non-CPAP group (Fig. 1). The proportion of males was higher in the CPAP group, while the distribution of age was similar (Table 3).

**Table 3:** Patient demographics and presumed cause of ARF assessed by the EMS

	CPAP (*N* = 171)	Non-CPAP (*N* = 739)	*ρ*-value
Patient demographics			
Male, %	60 %	50%^a^	0.02
Age, years, median (IQR)	73 (64–80)	74 (64–81)^b^	0.99
Presumed cause of ARF			
Acute cardiopulmonary oedema	18.1 %	13.3 %	0.10^c^
Asthma, acute exacerbation	5.3 %	7.3 %	0.34^c^
COPD, acute exacerbation	57.3 %	45.5 %	0.01^c^
Combined COPD and acute cardiopulmonary oedema	2.3 %	3.4 %	0.63^d^
Combined COPD and asthma	1.2 %	4.2 %	0.07^d^
Other	12.9 %	14.2 %	0.65^c^
Unknown or not reported	2.9 %	12.2 %	<0.01^d^

No missing data unless otherwise stated

*ARF* acute respiratory failure, *COPD* chronic obstructive pulmonary disease, *CPAP* continuous positive airway pressure, *EMS* emergency medical services, *IQR* interquartile range

^a^ *N* = 716

^b^ *N* = 704

^c^Calculated by chi-square test

^d^Calculated by Fisher’s exact test

Presumed causes of acute respiratory failure assessed by the EMS varied between the two study groups; acute exacerbation of COPD and acute cardiopulmonary oedema were more frequently reported in the CPAP group whereas unknown causes were more frequent in the non-CPAP group (Table 3). Amongst “other” causes were e.g. unspecified dyspnoea, angina pectoris, allergic reactions and unconsciousness. On arrival at scene, patients in the CPAP group had a lower SpO_2_ (87 % versus 92 %) and a higher respiratory rate (32 breaths/min versus 28 breaths/min) than non-CPAP patients, while blood pressures were similar (Table 4).

**Table 4:** Vital signs recorded upon arrival at scene and arrival at hospital

	CPAP	Non-CPAP	*ρ*-value
Status on arrival at scene			
SpO2, %	87 (77–94)	92 (85–97)	<0.01
	(*N* = 159)	(*N* = 723)	
Respiratory rate, breaths/min	32 (28–38)	28 (24–32)	<0.01
	(*N* = 163)	(*N* = 692)	
Systolic blood pressure^a^, mmHg	157 (± 34)	155 (± 33)	0.51
	(*N* = 159)	(*N* = 704)	
Diastolic blood pressure^a^, mmHg	92 (± 24)	93 (± 24)	0.93
	(*N* = 145)	(*N* = 704)	
Status on arrival at hospital			
SpO2, %	96 (94–99)	96 (91–98)	0.02
	(*N* = 168)	(*N* = 715)	
Respiratory rate, breaths/min	25 (21–30)	24 (21–30)	0.67
	(*N* = 163)	(*N* = 550)	
Systolic blood pressure^a^, mmHg	140 (± 25)	142 (± 30)	0.25
	(*N* = 156)	(*N* = 679)	
Diastolic blood pressure^a^, mmHg	85 (± 19)	86 (± 24)	0.59
	(*N* = 145)	(*N* = 676)	

Data presented as median (IQR) unless otherwise stated

*CPAP* continuous positive airway pressure, *IQR* interquartile range, *SD* standard deviation, *SpO*_*2*_ peripheral capillary oxygen saturation

^a^mean (± SD)

### Effectiveness of prehospital CPAP versus non-CPAP treatment

**Fig. 2:**
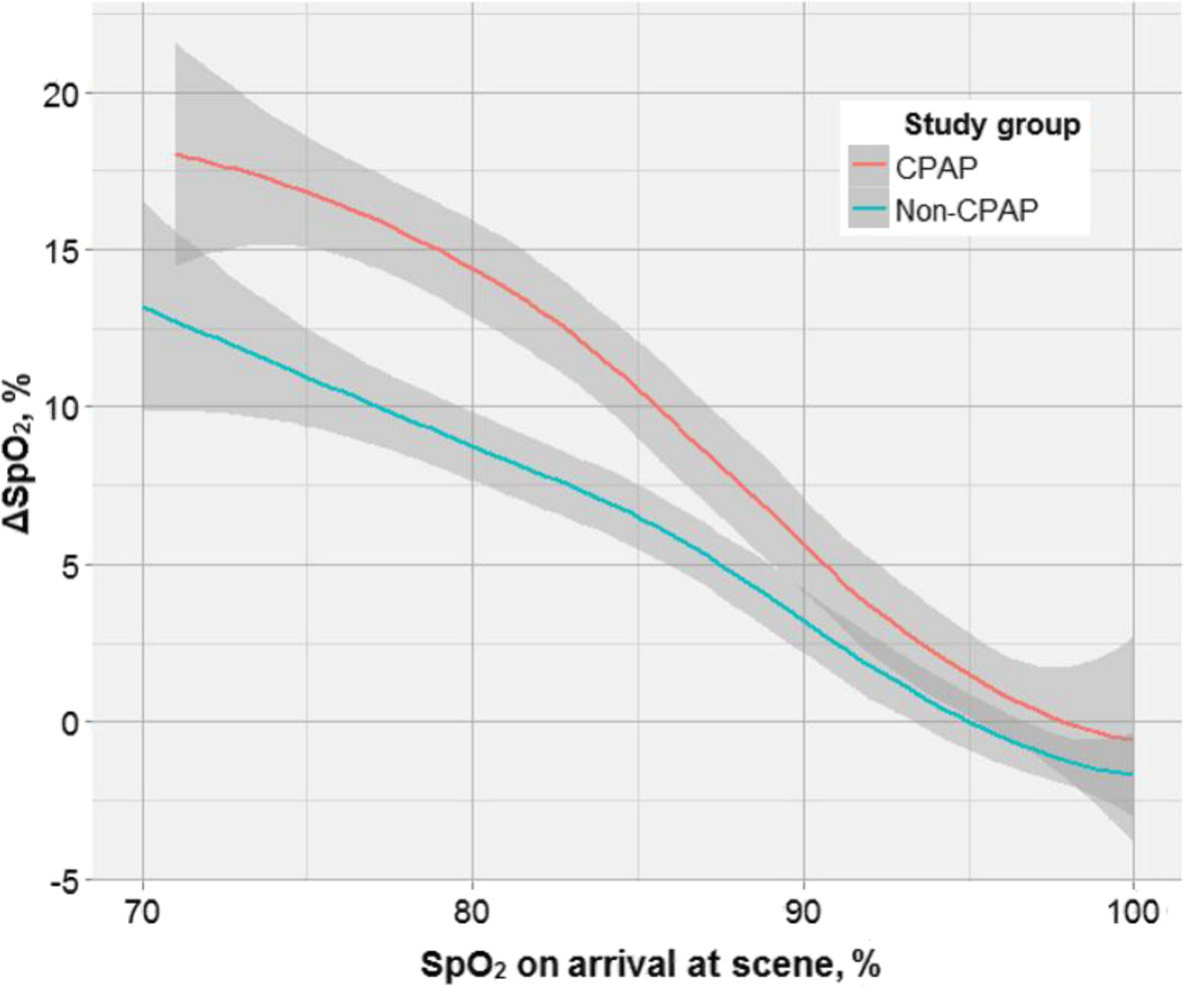
Changes in SpO_2_ from arrival at scene to arrival at hospital as a function of initial SpO_2_ value and according to study group. Grey areas represent 95 % confidence limits. Number of points: 30. CPAP group: *N* = 136. Non-CPAP group: *N* = 663

During transport, CPAP patients had a larger median increase in SpO_2_ compared to non-CPAP patients (8 percentage points versus 2 percentage points, *p* < 0.01, 95 % CI [4, 7]) (CPAP group: *N* = 158, non-CPAP group: *N* = 712). Increases in SpO_2_ were higher the lower the initial SpO_2_ value as demonstrated in Fig. 2, and this pattern was unaffected by adjustment for sex and age (Table 5).

**Table 5:** Changes in SpO_2_ during transport to hospital

	SpO_2_ on arrival at scene					
	71–80 %		81–90 %		>90 %	
	CPAP	Non-CPAP	CPAP	Non-CPAP	CPAP	Non-CPAP
Unadjusted	+16.2*	+9.4*	+9.8*	+5.9*	+1.5	0
Adjusted for sex and age	+16.5*	+9.2*	+9.9*	+6.0*	+1.6	−0.1

Changes in SpO_2_ from arrival at scene to arrival at hospital according to initial SpO_2_ value and study group

CPAP group: *N* = 136. Non-CPAP group: *N* = 663

*CPAP* continuous positive airway pressure, *SpO*_*2*_ peripheral capillary oxygen saturation

*p < 0.05

In this linear model, initial SpO_2_ ≤70 % were excluded due to imprecision of the measuring device; 23 in the CPAP group and 60 in the non-CPAP group. The decrease in respiratory rate was larger among CPAP patients than among non-CPAP patients; a median decrease of 8 breaths/min versus 2 breaths/min, respectively (*p* < 0.01, 95 % CI [−5, −3]) (CPAP group: *N* = 162, non-CPAP group: *N* = 539). On arrival at hospital, SpO_2_ (CPAP 96 % versus non-CPAP 96 %) and respiratory rate (CPAP 25 breaths/min versus non-CPAP 24 breaths/min) did not differ between the study groups (Table 4). Lowest respiratory rate after CPAP treatment was 11 breaths per minute.

### Concurrent prehospital management in CPAP versus non-CPAP groups

Use of concurrent pharmacological treatments varied between the two study groups (Table 6); a larger proportion of patients in the CPAP group received furosemide, fentanyl and nitroglycerine than in the non-CPAP group, while a smaller proportion received salbutamol. One patient (0.6 %) in the CPAP group and eight patients (1.1 %) in the non-CPAP group were intubated before arrival at hospital (missing data in 0 patients).

**Table 6:** Prehospital pharmacological treatment

	CPAP (*N* = 171)	Non-CPAP (*N* = 739)	*ρ*-value
Intravenous			
Furosemide, N (%)	50 (29 %)	152 (21 %)	0.01
Fentanyl, N (%)	29 (17 %)	88 (12 %)	0.08
Sublingual spray			
Nitroglycerine, N (%)	46 (27 %)	140 (19 %)	0.02
Inhalation			
Salbutamol, N (%)	108 (63 %)	589 (80 %)	<0.01

No missing data

*CPAP* continuous positive airway pressure

## Discussion

In this prospective study of prehospital CPAP to patients with acute respiratory failure, adherence to treatment was high as 88 % of all patients were treated with CPAP during the entire transport from scene to hospital. Adverse events were minor, except for one serious event, a pneumothorax. CPAP as a supplement to standard care was associated with larger improvements in SpO_2_ and respiratory rate compared to a historical cohort of patients treated with standard care alone.

### Adherence to treatment

CPAP is used in-hospital to treat patients with acute cardiopulmonary oedema, but is not recommended for acute exacerbations of COPD or asthma. Previous studies of prehospital CPAP treatment with cohorts of 60–149 patients with acute cardiogenic pulmonary oedema have not reported any serious adverse events [9–15]. Williams et al. reported that only 41 % of patients, who were coded as acute pulmonary oedema by a paramedic had this particular emergency department discharge diagnosis [26]. Accordingly, a CPAP protocol restricted to patients with presumed acute cardiopulmonary oedema is likely to be started even if the patient is suffering from acute respiratory failure from a different aetiology. In our study, we investigated if CPAP was safe to use in prehospital patients with acute respiratory failure from either acute cardiopulmonary oedema, acute exacerbation of COPD or asthma. Prehospital data collection including potential adverse events was planned prospectively, which made paramedics consistently assess and report any adverse events, minor as well as serious. Potential serious adverse events of prehospital CPAP treatment include apnoea, therapy failure in cardiogenic shock for example due to acute myocardial infarction and risk of either resulting in a pneumothorax or worsening an existing one [27, 28]. In the present study, no patients developed a systolic blood pressure <80 mmHg as an indication of impaired cardiac function. Likewise, no patients developed apnoea, yet two patients experienced worsening dyspnoea upon treatment with CPAP. We consider it likely, that the one serious adverse event of a pneumothorax in our cohort was due to or worsened by CPAP. A similar adverse event has been reported in a case series study, where one patient had failed prehospital CPAP and a pneumothorax was discovered following intubation and mechanical ventilation in the emergency department [29]. In five cases in our study, SpO_2_ actually decreased upon CPAP treatment, indicating a worsening of the condition. All adverse events of CPAP treatment in the present study was managed and treated adequately in the prehospital setting with shift to standard care or assistance from a physician staffed MECU. These cases illustrate how CPAP may be administered inadvertently to a patient, whose final diagnosis is different than presumed by paramedics. Our results demonstrate, that CPAP treatment is feasible in a prehospital setting, where time is limited and differentiation between cardiogenic, pulmonary and other aetiologies of acute respiratory failure is difficult. The results also emphasize the need for caution and frequent reassessment of the patient’s SpO_2_ and clinical condition during CPAP treatment and call for physician assistance to manage any complications.

### Effectiveness

The present study was too small to allow assessment of any effect of prehospital CPAP on mortality, nor could the effectiveness of CPAP treatment in specific disease conditions be examined. The study design, however, reflects the daily prehospital working environment, in which EMS personnel generally do not give specific diagnoses before considering how to treat the patient, and on average respiratory parameters improved in our study population. As expected from the oxygen-haemoglobin dissociation curve, the effectiveness of treatment on SpO_2_ reported in this study diminished with increasing initial SpO_2_ values (>90 %), which should be taken into consideration when planning future studies. Measures of gas exchange would have been valuable to the study. Originally, registration of end-tidal CO_2_ was mandatory; however, the variable was dropped as the patients’ CO_2_ were diluted from the high gas flow in the CPAP system. Development of respiratory acidosis as an outcome measure was not applicable in this study as the patients’ arterial gas samples were not routinely entered in to the patients’ electronic medical records. In future studies, arterial gas sampling will be a useful outcome measure. Addition of prehospital CPAP to medical therapy improved SpO_2_ and respiratory rate in patients with acute respiratory failure in several observational studies [16, 27, 28]. Comparison of standard medical therapy plus CPAP to standard medical therapy alone has shown, that CPAP is superior when using SpO_2_ or respiratory rate as outcome measures of effectiveness in patients with acute cardiopulmonary oedema or acute respiratory failure [10, 12, 17]. Two recent studies of prehospital patients with respiratory distress, conducted in North American urban settings, did not find any benefit of CPAP when comparing groups before and after implementation [30, 31]. The study protocols aimed at patients with presumed heart failure, COPD or asthma and the study patients’ initial oxygen saturation and respiratory rate were comparable to initial values in this study population. Transport time from departure from scene to arrival at emergency department was considerably shorter than in the present study, only 9.6 min [30]. Distance and transport time to hospital is an important factor when deciding how to treat patients with acute respiratory failure during ambulance transport; it is always a balance between time used to initiate a prehospital treatment and a faster arrival to definitive treatment at hospital. Our study showed that CPAP was effective in an EMS system with a mean treatment time of 35 min, reflecting the relatively long transport times in the region. In the prehospital setting, oxygen therapy should be given cautiously; high flow oxygen has been associated with increased mortality compared to titrated oxygen treatment in patients with presumed acute exacerbation of COPD [32]. In both our study groups, the high concentration of oxygen may have removed nitrogen from the alveoli and subsequently induced absorption atelectasis and it may also have led to hypercapnia in oxygen sensitive COPD patients. We presume that no patients experienced severe hyperoxaemia and/or severe hypercapnia in our study, since the two examples of decrease in level of consciousness were minor and respiratory rate did not decrease below 11 breaths per minute following CPAP treatment. Moreover, in acute myocardial infarction, prehospital oxygen therapy has been associated with larger myocardial infarct size compared to no oxygen therapy in patients with acute ST-elevation myocardial infarction and no hypoxia [33]. Lower fractional oxygen with CPAP improved oxygen saturation and respiratory rate in patients with acute respiratory failure in previous observational studies [17, 27]. Nonetheless, both our study groups were treated with 100 % oxygen as titrated oxygen treatment was not yet possible in the region’s ambulances.

### Strengths and limitations

Strengths of the present study include a population based and consecutive sample of 171 patients treated with CPAP in daily prehospital practice, and few patients were lost to follow-up. External validity of this observational study is regarded as high in countries with similar disease patterns and EMS structures. The study design contains limitations; retrospective data collection from the historical non-CPAP group, risk of regression to the mean value and obvious confounding in the form of concurrent pharmacological treatment. No changes, apart from addition of CPAP trough a tight facemask, were made to EMS treatment for acute respiratory failure across the two study periods. Nevertheless, concurrent pharmacological treatments varied between study groups, probably due to differences in the case mix between the two groups, and though differences were small, the impact on our results is unknown. During the CPAP study period, inclusion was limited to cases, in which a paramedic vehicle was available and dispatched, causing a selection bias. In the study design, the individual paramedic both served as treatment provider and data collector by which observer bias was induced. We cannot estimate the impact of case mix differences that were not included in the linear model, e.g. presumed pathology, initial respiratory rate or any bias resulting from the varying selection criteria of patients in the two study groups. Due to above mentioned limitations, the presented results ought to be interpreted as exploratory results. As patients with initial SpO_2_ values ≤70 % were excluded from both treatment groups due to imprecise measurement, we are not able to conclude on effectiveness of CPAP in patients with SpO_2_ values below this limit.

## Conclusions

In a three-tier EMS system including on-call physician backup, adherence to prehospital CPAP treatment administered by paramedics was high in patients with acute respiratory failure. Few adverse events were reported, including one potentially serious adverse event averted by a rendezvous physician. Patients treated with standard care plus CPAP experienced larger increases in SpO_2_ and reduced respiratory rate during prehospital transport compared to a historical cohort of patients treated with standard care only. The study is useful in future trial planning and for EMS systems intending to implement prehospital CPAP. Randomised studies are required to demonstrate short-term and long-term effects of prehospital CPAP including effects of low versus high flow oxygen.

## Abbreviations

amPHI^TM^: Ambulance pre-hospital intervention (trademark)
COPD: Chronic obstructive pulmonary disease
CPAP: Continuous positive airway pressure
EMCC: Emergency medical communication centre
EMS: Emergency medical services
ICU: Intensive care unit
MECU: Mobile emergency care unit
NIV: Non-invasive ventilation
PEEP: Positive end-expiratory pressure
SpO_2_: Peripheral capillary oxygen saturation
